# Analysis of single comments left for bioRxiv preprints till September 2019

**DOI:** 10.11613/BM.2021.020201

**Published:** 2021-04-15

**Authors:** Mario Malički, Joseph Costello, Juan Pablo Alperin, Lauren A. Maggio

**Affiliations:** 1 Meta-Research Innovation Center at Stanford (METRICS), Stanford University, San Francisco, USA; 2 Uniformed Services University of the Health Sciences, Bethesda, Maryland, USA; 3 Scholarly Communications Lab, Simon Fraser University, Vancouver, British Columbia, Canada; 4 School of Publishing, Simon Fraser University, Vancouver, British Columbia, Canada

**Keywords:** preprint, preprints as topic, comment, peer review, scientific misconduct

## Abstract

**Introduction:**

While early commenting on studies is seen as one of the advantages of preprints, the type of such comments, and the people who post them, have not been systematically explored.

**Materials and methods:**

We analysed comments posted between 21 May 2015 and 9 September 2019 for 1983 bioRxiv preprints that received only one comment on the bioRxiv website. The comment types were classified by three coders independently, with all differences resolved by consensus.

**Results:**

Our analysis showed that 69% of comments were posted by non-authors (N = 1366), and 31% by the preprints’ authors themselves (N = 617). Twelve percent of non-author comments (N = 168) were full review reports traditionally found during journal review, while the rest most commonly contained praises (N = 577, 42%), suggestions (N = 399, 29%), or criticisms (N = 226, 17%). Authors’ comments most commonly contained publication status updates (N = 354, 57%), additional study information (N = 158, 26%), or solicited feedback for the preprints (N = 65, 11%).

**Conclusions:**

Our results indicate that comments posted for bioRxiv preprints may have potential benefits for both the public and the scholarly community. Further research is needed to measure the direct impact of these comments on comments made by journal peer reviewers, subsequent preprint versions or journal publications.

## Introduction

The practice of sharing preprints, authors’ versions of non-peer reviewed manuscripts, is on the rise. Once almost exclusively limited to the fields of high energy physics and economics on arXiv, RePec and SSRN preprint servers, preprints have gained much ground across a wide range of disciplines, including medical biochemistry and laboratory medicine (*1*). Preprints are also increasingly indexed in large scholarly databases and search engines (*e.g.*, PubMed, Crossref, Lens, Dimensions, Microsoft Academic), and major manual referencing styles have issued guidance on how preprints should be cited in scholarly papers (*2*, *3*). Meta-research on preprints, however, remains scarce and is mostly limited to the explorations of two servers: arXiv (which includes sections on biomolecules and genomics) and bioRxiv (which includes sections on biochemistry and genomics). This limited research has shown that citation of preprints in scholarly literature had increased, and that articles first posted as preprints had higher citations rates and Altmetric scores than those not posted as preprints (*2*). Additionally, only minimal changes were found between preprints and the versions (of record) published in journals (*4*).

In 2020, the COVID-19 pandemic led to a large increase in the posting of preprints, as well as scrutiny and the number of comments they received on both social media platforms (*e.g.*, Twitter) and comment sections of servers on which they are posted, with some comments prompting preprint retractions (*5*, *6*). However, despite 70% of preprint servers allowing users to post comments on their platforms, and researchers perceiving the possibility of receiving comments as one of the advantages of preprints compared to traditional publishing, no research, to the best of our knowledge, has examined the nature of comments or actors involved in preprint commenting (*1*, *2*, *7*). In this study, which originated before the COVID-19 pandemic, we aimed to conduct an exploratory analysis of type of comments left on the bioRxiv servers. Furthermore, at that time, the majority of preprints with comments only had a single public comment, and so we decided to focus exclusively on those comments.

## Materials and methods

We conducted a cross-sectional study of bioRxiv preprints that received a single comment on the bioRxiv platform between 21 May 2015 (the earliest date available through the bioRxiv comment Application Programming Interface - API) and September 9, 2019 (study data collection date).

### Data collection

As part of our *Preprint Observatory* project, we collected all available comments and related metadata using the bioRxiv comment API (*8*, *9*). Collected data included DOIs, links to the preprints, commenter username (*i.e.*, proper name or created name), date and time the comment was posted, and the comment text. Data was stored and managed in Microsoft Excel (Microsoft, Redmond, USA) and covered 6454 comments posted for 3265 unique preprints (which represented 6% of 56,427 preprints deposited on or before 9 September 2019). However, during data analysis, we realized, that the bioRxiv comment API did not provide access to comments posted before May 2015, so the percentage of commenting is probably slightly higher, but based on the data we had, it is likely that less than 10% of all preprints received comments on the bioRxiv website). Of the 3265 preprints in our database, 1983 (60%) received only a single public comment, and we decided to focus on them in this study. We enriched the data of those 1983 comments by adding preprint authors, subject area classification, word count for comments, and published date of preprints as reported in Crossref and extracted during our *Preprints Uptake and Use Project* (*10*). Finally, we classified the commenters as authors of the preprints or non-authors, and for authors we also captured their byline order (*i.e.*, first, last or other – defined as neither first nor last).

### Data analysis

Comments’ were inductively classified by using an iterative process of reading the text, open coding and constant comparison (*11*). The initial comment types were devised by an analysis conducted on a sample of 35 comments, and later expanded using a sample of 200 comments. This initial categorization revealed distinct differences in the content of comments left by authors of the preprints and those left by non-authors.

### Identity of the commenter

We first checked whether each comment had been posted by an author of the preprint. This was done by comparing if the posted username matched any of the names of the preprint authors (and was helped by a simple full username search with any of the authors’ names - the simple search detected only 301 out of our later manually detected 617 cases as usernames often contained initials or symbols that were not an exact match with the names used in the preprint author byline). If the username was a pseudonym or a lab name, we classified the commenter as a non-author. During coding, we amended our initial classification if the comments’ contents provided identification of the commenter.

### Content analysis

After grouping comments by the commenter type (author or non-author), three of us independently categorized all comments. Each comment could be classified to multiple categories. The only exception to this rule was if the comment was similar in structure and content to a full peer review report that is traditionally submitted as part of a journal peer review process. In those cases, we decided not to analyse the full contents of such reviews as they were often authored by multiple authors, contained multiple review reports, or included links to detailed reports posted on other websites. For all other comments, we classified the type of content they contained, but not the number of instances of each type they contained. For example, if the content type was a suggestion, we did not count the number of suggestions made in the comment, *i.e.*, one suggestion for formatting a table, another for a figure, and an additional suggestion for expanding the literature section. The three coders held weekly meetings online after coding batches of 200 to 300 comments. These meetings allowed for comparison of categorizations, resolving differences, clarification of existing or introduction of new categories. Before each meeting, we would compare differences between the coders. If only one coder categorized a comment differently (*e.g.*, did not mark a specific category) we re-read the comment, ruled on the found difference, and recorded the final categorization in the main database. When a single coder indicated a category the other two did not, or all coders disagreed on the categorization, the comment was marked and discussed at a weekly meeting until consensus was reached. We observed that our initial disagreement was most common for comments we categorised as suggestions or criticisms, and where tone, rather than content, dictated the categorisation (*e.g.*, Comment 1: *“Great to see more well assembled lizard genomes, but it would have been nice to cite the more recent assemblies of…”;* Comment 2: *The authors state in the introduction that* [method] *has not been yet been reported”. I beg to differ… following models have been generated and published*… [provides references to 3 studies]. We categorised comment 1 as suggestion, and comment 2 as criticism, based on their tone even though they both provided authors with additional references. As comments could have multiple categories, comment 1 was also classified as a praise).

While methods exist for calculating inter-rater reliability for data that could be classified as belonging to multiple categories, after each weekly meeting we only stored our agreed upon classification, so we cannot reconstruct the initial disagreements to produce such rating. It was also not our goal to study the difficulty of classifying comments, but rather, using a consensus approach, to explore the different types of comments posted on bioRxiv (before the pandemic). Our final classification tree and an example comment for each category are shown in Supplementary Table 1, and all comments and our assigned categories in our project’s database (*8*). Finally, to see if comments of preprints that received a single public comment, and that were the focus of our study, differed from first comments left for preprints which received more than one comment, we also randomly chose 200 of the latter preprints and analysed their first comments. This sub-analysis showed that all of these comments could be classified under our identified comment types.

### Statistical analysis

We report absolute numbers and percentages for types of comments, and medians and interquartile ranges (IQR) for number of words per comment, number of comments per preprint and days from posting of the preprint to the comment. As number of words and days are integers, when medians or 25^th^ and 75^th^ percentiles had decimals, we rounded them to ease readability. Note on word count: As the texts of the comments were retrieved in HTML syntax, we replaced the hyperlink syntax (*e.g*., <a….a>) with the word LINK and counted it as only one word. When references were written out as *Author et al., year*, or *PMID: number*, those were counted by as many words as were written. Differences in number of words and time to publication between author and non-author comments were tested with Mann-Whitney test. We did not use time-to-event analysis as information for comments posted before May 2015 was not available through the API. Analysed comments came from all 27 bioRxiv subject area classifications (assigned by the authors during preprint upload, Supplementary Table 2). Even though there were slight differences in the number of comments per subject area, we chose not to explore those differences for several reasons. The sample size was too small for such an analysis, and perceived preprint impact, as well as authors’ prestige, country and other factors might influence the posting of comments (and those were not available to us). Significant differences were considered for P < 0.05. All analyses were conducted using JASP version 0.12.2. (https://jasp-stats.org/).

## Results

Between 21 May 2016 and 9 September 2019 there were 1983 bioRxiv preprints that received a single public comment on the bioRxiv website. More than two thirds of those comments were posted by non-authors (N = 1366, 69%), while the remainder were posted by the preprint’s authors themselves (N = 617, 31%, Table 1). Overall, the non-author comments were longer than comments posted by the authors (Mann-Whitney test, P < 0.001), and they were posted a median of 23 days after the preprints. In comparison, authors’ comments were posted after a median of 91 days (Mann-Whitney test, P < 0.001). Differences between types of comments, with regards to number of words and days between preprint and comment publication, are shown in Table 1.

**Table 1:** Type of comments, word count and days lapsed until comments were made on bioRxiv for preprints that received a single public comment between 21 May 2016 and 9 September 2019

Comment type*	**N (%)^†^**	**Words^‡^**	**Days^‡^** **(from preprint to comment)**
**Non-author’s comment**	1366 (69)	38 (17-83)	23 (3-117)
Praise	577 (42)	36 (17-70)	13 (2-93)
Suggestion	399 (29)	46 (26-80)	14 (2-90)
Criticism	226 (17)	78 (41-148)	21 (3-104)
Asking for clarification	213 (16)	41 (22-73)	31 (4-134)
Full peer review report	168 (12)	397 (78-785)	46 (13-118)
Issue detected	132 (10)	22 (10-38)	18 (2-80)
Asking for raw data or code	41 (3)	29 (13-52)	21 (2-97)
Publication status update	34 (2)	12 (10-15)	226 (96-343)
Other	90 (7)	20 (11-38)	20 (3-128)
**Author’s comment**	617 (31)	18 (11-32)	91 (3-238)
Publication status update	354 (57)	17 (10-20)	178 (85-293)
Additional study information	158 (26)	21 (12-35)	10 (2-121)
Soliciting feedback	65 (11)	21 (14-35)	2 (1-8)
Study promotion	44 (7)	23 (14-63)	1 (0-3)
Reply or thanks for received comments	29 (5)	45 (22-111)	21 (5-98)
Other	41 (7)	28 (17-48)	2 (1-17)
Words and days from preprint to comment are presented as median (interquartile range). *Examples of comment types can be found in Supplementary Table 1. ^†^Percentages do not add up to a 100, as comment’s content could contain more than one comment type. ^‡^The median and IQRs for number of words in this table are calculated based on the total number of words of a comment, not its individual parts (*i.e.*, if a comment contained both a praise and a suggestion, its total word count is added to both of those categories).			

Twelve percent of non-author’s comments (N = 168) were full review reports resembling those traditionally submitted during the journal peer review process. They were authored by either single individuals or group of authors (Supplementary Table 3). The latter most commonly published their review following a journal club discussion. Comments not resembling full peer review reports most commonly praised the preprint (N = 577, 42%), made suggestions on how to improve it (N = 399, 29%), or criticized some aspect of the preprint (N = 226, 17%) (Table 1). Praise was most commonly found alongside suggestions or comments asking for clarifications, and least commonly alongside comments that criticised the preprint, reported issues or that inquired of the preprints publications status (Supplementary Table 4). Praise words alone (*e.g*., “*Amazing work!”*) constituted 6% (N = 86) of comments. Comments containing suggestions often included suggestions of literature (co-)authored by the commenter or suggestions of other literature (Supplementary Table 3).

Lastly, we present some examples of the comments we classified as belonging to the “other” category (a full list of those comments is available on our project website). There were three comments that raised research integrity issues (a possible figure duplication, an undeclared conflict of interest, and use of bots to inflate paper download numbers). There were also comments that raised personal issues. In one comment a parent requested more information on a rare disease (covered by the preprint) that was affecting their children, and in another case an individual inquired about possible PhD mentors for a topic related to the preprint. There were also comments that touched upon the culture of preprinting, with one comment asking authors to include brief summaries of what had changed between preprint versions, another expressing a view that preprints make traditional publishing redundant, and one praising authors for replying to questions they asked through email. Similarly, one comment we classified as full peer review report, also included a statement of hope “*to get more comments on bioRxiv*…*prior to submission to a peer reviewed-journal”* as they would “*rather have a revised pre-print than a correction / retraction” in a journal*.

Authors’ comments most commonly contained updates about the preprint’s publication status (N = 354, 57%), additional information on the study (N = 158, 26%), or solicited feedback for the preprint (N = 65, 11%, Table 1). Of all authors’ comments, most were posted by the first author of the preprint (we could not identify the byline order for four percent of comments, N = 22, as the registered username was either a pseudonym, *e.g*., *W1ndy*, or a lab name, *e.g*., *Lewy Body Lab*). A small percentage (N = 29, 5%) of author comments were replies to feedback authors received elsewhere, *e.g*., during peer review or through personal emails (Supplementary Table 5). Lastly, as above, we present few examples of authors’ comments classified as belonging to the “other” category (with full list of those available on our project website). In five comments authors requested suggestions on where to publish their preprint, and in one comment authors mentioned that an editor saw their preprint and invited them to submit it to their journal. In one comment, an author alerted the readers of an error in a figure and also playfully chided (using a smiley emoticon) the co-author for hastily uploading the files before checking them. In another, co-authors alerted readers that the preprint had been posted without the approval of the co-authors and urged the scientific community to ignore this version (to date the preprint in question has not been retracted). Finally, in one example (of a comment classified as a publication status update), the author said they did not plan to submit the preprint to a journal, as publishing on bioRxiv makes it freely available to everyone.

## Discussion

Our analysis of single comments left for bioRxiv preprints before September 2019 found that more than two thirds of those comments were left by non-authors and were most commonly praises, suggestions, or criticisms of the preprints. Additionally, almost a sixth of non-author comments contained detailed peer review reports akin to those traditionally submitted during the journal peer review process. Despite, to the best of our knowledge, our study being the first to analyse comments left on preprint server’s website, these findings support previous studies that showed the opportunity to receive feedback was perceived as one of the benefits of preprints compared to traditional publishing (*2*, *7*). However, we also found that less than ten percent of all bioRxiv preprints received public comments before the COVID-19 pandemic. This low prevalence of scholarly public commenting has been previously observed for post-publication commenting of biomedical articles, and was the reason for discontinuing PubMed Commons, the National Library of Medicine’s commenting resource (*12*). Similar low prevalence of post-publication commenting has also been found across disciplines on PubPeer (*13*). Nevertheless, as has been previously stated for those services, some of those comments have been crucial for scholarly debates and even led to retractions of papers, a practice also observed for bioRxiv preprints (*12*-*14*). In our study, we observed that eleven percent of authors’ comments were actively inviting others to comment on their preprint, with one comment explicitly stating that they would rather make changes to the preprint than to a version published in a journal.

The lack of traditional peer-review is often perceived as the biggest criticism of pre-printing, alongside cases of information misuse and posting of low-quality studies (*15*). Thus, bioRxiv (alongside arXiv and medRxiv) have displayed clear disclaimers for COVID-19 preprints that state preprints are “*preliminary reports that have not been peer-reviewed”* and they should not be “*reported in media as established information*” (*16*). Related to this criticism and the benefits of preprint commenting, there has also been a rise of specialised preprint review services (*e.g*., PreReview, Review Commons, Peerage of Science) or overlay journals (*e.g*., Rapid Reviews, JMRIx) aimed at providing expert reviews for preprints, or endorsement of preprints *(e.g*., Plaudit) (*17*-*22*). Additionally, in December 2020, journal eLife announced they would only be reviewing papers that have been first posted as preprints, and that they are switching from being a publisher to “an organization that reviews and certifies papers that have already been posted” as preprints (*23*). On a similar note, to emphasize the possible role that commenting has in the scientific discourse, reference software Zotero can display references that have PubPeer comments, and a recently launched biomedical search engine PubliBee, implemented (up)voting of comments (*24*, *25*). Upvoting of comments is already available for several preprint servers, including bioRxiv, that utilize Discus as the commenting platform (*26*). It will be interesting to see if more journals and publishers implement similar changes, and if the focus on reviewing preprints will lead to a decrease in the practice of (double)blind review of manuscripts.

Alongside posting of full peer review reports, our study also confirmed other known practices and potential benefits associated with the preprinting culture. For example, using preprints as final publication outputs, soliciting or being invited by editors to publish studies posted as preprints, calling out suspected research integrity issues, engaging in discussion or proposing collaborations, as well as publishing of peer review reports from those training on how to conduct peer review, or from journal club discussions. These findings may provide authors encouragement to consider or continue depositing preprints.

Furthermore, we have shown that almost a third of the comments were left by the authors of the preprints, and their comments were mostly updates of preprints’ publication status or additional information about the studies. Authors’ comments were also in general left after a much longer period than those of the non-authors. This aligns with found median times of 166 to 182 days between posting a preprint on bioRxiv and publication of that study in a journal, which were similar to the median time of 172 days we found for comments on publication status updates (*2*).

Despite being the first analysis of single comments posted for bioRxiv preprints, our study is not without limitations. We did not attempt to define if non-authors that posted comments were indeed peers, nor did we compare their expertise or publication records with those of the authors of the preprint on which they were commenting. We are also aware that some comments were left by patients, students and the individuals that stated a lack of expertise in the field. However, defining and soliciting feedback from a competent peer is known to be difficult, with previous studies demonstrating minimal agreements between peers assessing the same study (*27*, *28*). Furthermore, we did not attempt to define the quality of the comments, nor if the contents of comments (*e.g*., raised criticisms or suggestions) were indeed valid. We also did not check if comments led to changes or updates of the preprints or eventual published manuscripts, nor if the authors were even aware of them. Regarding the latter, as we analysed preprints that only had a single comment, none of the authors used the preprint platform to reply to them. We however did find that five percent of authors’ comments were replies to comments or peer reviews they received elsewhere, and we did encounter an example of a non-author comment that indicated they communicated with the authors by email. The purpose of our research was not to provide external validity of the claims stated in the comments, but rather showcase, for the first time, the most common types of comments left on the platform (before the COVID-19 pandemic). Our study is also limited in that we did not analyse discourse that might occur in preprints which received multiple comments. However, we did analyse the first comments of a random sample of 200 of such preprints to confirm that they do fall within the categories analysed here. Finally, we acknowledge that our backgrounds are not in life sciences, and that this may have affected our ability to make a clear distinction between some comment types, especially in distinguishing between suggestions and criticisms. We however feel that the observed differences in the number of words between our identified comment types, as well as prevalence or praise which is more common for comments that contained suggestions than criticisms, provides support for our categorization.

In conclusion our study indicates that bioRxiv commenting platform appears to have potential benefits for both the public and the scholarly community. Further research could measure the direct impact of these comments on comments made by journal peer reviewers, later preprint versions or journal publications, as well as the feasibility and sustainability of maintaining and moderating commenting sections of bioRxiv or other preprint servers. Finally, we believe that user-friendly integration of comments from server platforms and those posted on social media (*e.g*., Twitter) and specialized review platforms would be beneficial for a wide variety of stakeholders, including the public, authors, commenters, and researchers interested in analysis of comments.
